# *Colletotrichum* Species Associated with Citrus: Disease Biology, Global Diversity, Pathogenicity, and Biosecurity Implications

**DOI:** 10.3390/jof12090687

**Published:** 2026-09-13

**Authors:** Weixia Wang, Niloofar Vaghefi, Peter K. Ades, Jacqueline Edwards, Pedro W. Crous, Paul W. J. Taylor

**Affiliations:** 1 Faculty of Science, The University of Melbourne, Parkville, VIC 3010, Australia; weixiaw@student.unimelb.edu.au (W.W.); vaghefin@unimelb.edu.au (N.V.); petera@unimelb.edu.au (P.K.A.); 2 School of Applied Systems Biology, La Trobe University, Bundoora, VIC 3083, Australia; jackyedwards@live.com; 3 Westerdijk Fungal Biodiversity Institute, Uppsalalaan 8, 3584 CT Utrecht, The Netherlands; p.crous@wi.knaw.nl

**Keywords:** biosecurity, citrus, *Colletotrichum*, disease biology, global diversity, pathogenicity

## Abstract

Citrus is an important fruit crop worldwide and *Colletotrichum* species are important pathogens of this crop. *Colletotrichum* species infect a wide range of citrus tissue such as leaves, twigs, flower petals, immature and mature fruit, causing substantial losses in yield, fruit quality, and marketability. This review is a critical assessment of the major *Colletotrichum* species associated with citrus diseases, their global diversity, distribution, and biosecurity implications. This review also identifies areas in need of further research. Thirty-nine *Colletotrichum* species have been reported in association with citrus globally. Most *Colletotrichum* species are plant pathogens, while several are saprobes or exhibit endophytic or latent lifestyles. They also vary in host range, aggressiveness, and environmental adaptation. Several species can infect citrus hosts or tissues beyond those from which they were originally isolated, while some also infect plants from other genera. The nature of infection by *Colletotrichum* species highlights the need to include non-wound inoculation in bioassays designed to assess the ability of *Colletotrichum* isolates to penetrate the cuticle and epidermis and cause infection. Accurate identification, pathogenicity testing, and rapid molecular detection are therefore important for disease management, quarantine inspection, and plant biosecurity risk assessment.

## 1. Introduction

The genus *Colletotrichum* represents important fungal plant pathogens globally and is also capable of causing disease in animals [1]. Currently, the classification system of *Colletotrichum* comprises 20 species complexes with more than 340 *Colletotrichum* species. While a few species can be host-specific, the majority infect a wide range of host species [1,2,3,4,5]. Until recently, the host range of *Colletotrichum* species encompassed over 760 plant species belonging to more than 30 plant genera in tropical, subtropical, and temperate regions [1,6,7]. Among the host species, dicotyledonous plants were the most frequently recorded, accounting for more than 77%, followed by monocotyledonous hosts (20%), gymnosperms (1.4%), and ferns and mosses, which were seldom recorded [1].

**Figure 1:**
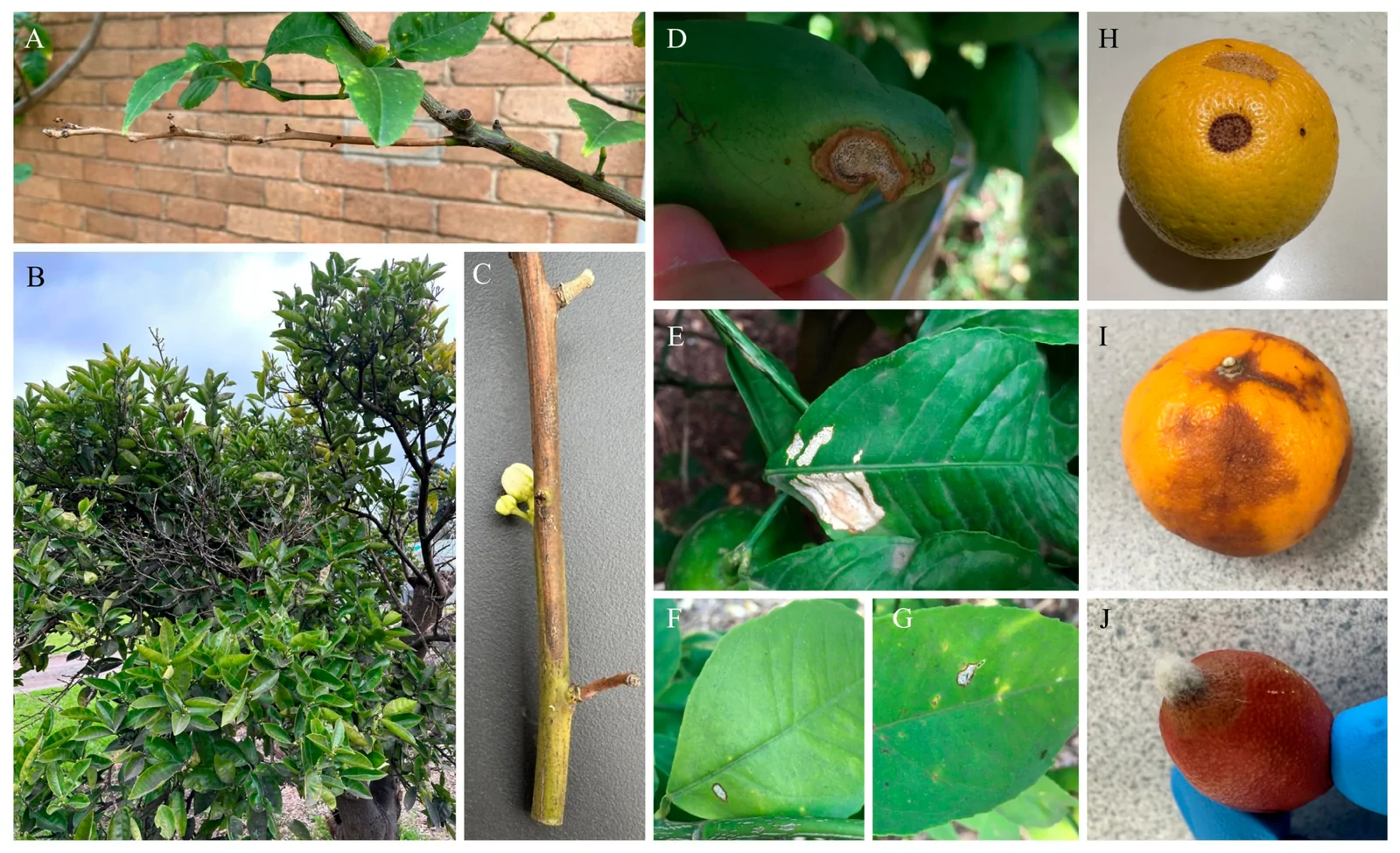
Disease symptoms on citrus twigs, leaves and fruits infected by *Colletotrichum* species. (**A**,**C**) Infected twigs of lemon (*Citrus limon*) showing necrotic lesions. (**B**) Infected twigs of orange (*Citrus* × *sinensis*) showing shoot dieback or withertip. (**D**,**E**) Infected orange leaves (*Citrus* × *sinensis*) with large necrotic lesions. (**F**,**G**) Infected leaves of lemon (*Citrus limon*) showing discrete small lesions. (**H**) Infected orange (*Citrus* × *sinensis*) fruit with a discrete necrotic lesion. (**I**) Infected mandarin (*Citrus reticulata*) fruit with tear-stain necrosis. (**J**) Infected finger lime (*Citrus australasica*) fruit with *Colletotrichum* hyphae emerging from a necrotic lesion.

*Colletotrichum* species infect *Citrus* leaves, twigs, flower petals, and cause disease on immature crops and fruit before harvest, as well as on mature crops and fruit products post-harvest, negatively affecting yield quantity, fruit quality and marketability (Figure 1) [2,8,9]. An epidemic of *Colletotrichum* in the field can easily lead to large losses in agricultural production and therefore cause significant economic losses for farmers [10]. *Colletotrichum* species are also isolated from healthy plant tissues and recognised as being in endophytic or latent life stages, or from dead plant tissues and recognised as saprobes [2,3]. Latent infection occurs when the pathogen enters the host tissue and remains inactive, then causes disease as the host’s physiology changes due to tissue maturation or exposure to abiotic stresses [2,3,11]. Latent infection is more frequently found in fruits, where the pathogen infects the fruit when it is immature and causes disease when the fruit starts to ripen, especially during the post-harvest period [12].

Citrus plants are perennial trees in the family Rutaceae that prefer to grow in areas with consistent sunlight and sufficient water [13]. Mandarin (*C. reticulata*), pomelo (*C. maxima*) and citron (*C. medica*) are the three ancestral species of the genus *Citrus*, and all commercially cultivated *Citrus* species are hybrids or introgressions between these three species [14,15]. As an important fruit crop worldwide, citrus is now mainly produced in Argentina, Australia, Brazil, China, Chile, Costa Rica, Egypt, the European Union, Guatemala, Israel, Japan, Mexico, Morocco, Peru, South Africa, Turkey, the United States, and Vietnam [16]. Grapefruit (*Citrus × paradisi*), lemon (*Citrus limon*), lime (*Citrus aurantiifolia*), tangerine/mandarin (*Citrus reticulata*) and orange (*Citrus × sinensis*) are the most popular species produced commercially [17]. Global citrus production for 2023/24 was estimated to be 102.6 million tons [16].

The high adaptability and wide host range of *Colletotrichum* species contribute to their persistence in citrus production regions. Accurate taxonomy of *Colletotrichum* species and knowledge of the pathogen’s pathogenic capacity on different hosts have important implications for plant biosecurity risk management and the development of integrated control strategies. Hence, this review aims to provide a critical assessment of the major *Colletotrichum* species associated with citrus diseases, their global diversity, distribution, and biosecurity implications, as well as to identify areas in need of further research.

## 2. Disease Cycle and Infection Strategies of *Colletotrichum* Species

*Colletotrichum* species have endophytic, latent or quiescent, hemibiotrophic, and necrotrophic lifestyles [2]. These fungi employ specialised infection strategies to infect host plants. Knowledge of the disease cycle and infection strategies of *Colletotrichum* species is essential for managing potential plant biosecurity risk and developing integrated control strategies.

*Colletotrichum* species favour warm temperatures between 25 °C and 28 °C with high air humidity, and become inactive under extremely low or high temperatures, low air humidity, or direct sunlight [2,18]. The pathogen survives unfavourable conditions, such as winter, by colonising plant seeds, twigs, leaf litter or alternative hosts. When environmental conditions become suitable, asexual fruiting structures (acervuli) are formed, which release conidiospores. However, in some species, sexual fruiting structures (perithecia) also develop and release ascospores. Released spores spread to nearby plant organs through rain splash, wind, or irrigation, germinate, and form infection structures (appressoria). Penetration pegs are formed from the appressoria, where high mechanical pressure is applied to enable penetration of the host cuticle and epidermal cell walls [19,20,21,22]. Some species in the acutatum complex have also been reported to enter leaves through stomata or wounds [23,24].

After successful penetration, most *Colletotrichum* species infect host plants through a hemibiotrophic strategy, which begins with a biotrophic phase and then transitions to a necrotrophic phase [2,20]. During the biotrophic phase, infection vesicles and primary hyphae develop inside the infected cells without causing necrosis. Eventually, the pathogen transitions to the necrotrophic phase, during which secondary hyphae develop and spread to adjacent cells, leading to host tissue necrosis [25].

*Colletotrichum* species employ a diverse range of infection strategies to restrain host defence mechanisms and facilitate colonisation [26]. The cuticle is an important physical barrier of plants that prevents initial infection [27,28]. Appressorium formation is an important strategy for most *Colletotrichum* species. Glycerol and other osmolytes accumulate in the appressorium, generating substantial turgor pressure of up to 8 MPa, which forces a narrow penetration peg to mechanically penetrate the host cuticle. Mutant *Colletotrichum* species that form fewer appressoria have been shown to have decreased pathogenicity [29]. The production of cutinase is also a critical strategy during the early stages of infection. Auyong et al. [30] found that *Colletotrichum truncatum* produced cutinase to assist the infection of chilli fruit and soybean leaves, and that the pathogenicity of the pathogen decreased when the cutinase gene was silenced [30]. After successful penetration, *Colletotrichum* species produce pathogen-encoded small secreted proteins (SSPs) to manipulate host metabolism or suppress defence signalling, thereby evading host immune responses during the initial biotrophic phase [31].

Another major defence mechanism of host plants is to enhance structural defence by reinforcing the cell wall, mainly through the accumulation of polysaccharides such as cellulose, hemicelluloses, and pectins [32]. In the citrus cell walls, lignin may accumulate after pathogen penetration to enhance resistance [27,33]. Gan et al. [34] found that *Colletotrichum gloeosporioides* and *C. orbiculare* produce a large number of carbohydrate-active enzymes (CAZymes), including cellulose-degrading and pectin-degrading enzymes, to degrade these polysaccharides and assist hyphal expansion [34].

Some *Colletotrichum* species remain dormant after entering plant tissue and cause disease once the host is under stress or environmental conditions become suitable for the pathogen to complete its life cycle [11]. The physiological maturity of the host can play an important role in disease development, as physiological and biochemical changes reduce defence activity and increase sugar content, thereby facilitating infection by *Colletotrichum* species [35,36,37].

Quiescence is frequently observed in *Colletotrichum* infections of fruit, in which fungal development is temporarily arrested until the host tissue ripens or reaches maturity. Although active growth is limited during this phase, the pathogen produces fungal effectors to modify the host environment and maintain its survival, which is similar to an endophytic lifestyle. *Colletotrichum gloeosporioides*, *C. acutatum*, *C. higginsianum*, *C. graminicola*, and *C. coccodes* actively release ammonium to increase the environmental pH, alter plant gene expression, modulate host responses, and induce confined cell death, after which the pathogen transitions to the necrotrophic phase [37,38]. The transcription factor PacC regulates genes encoding transporters, antioxidants, and cell-wall degrading enzymes and is crucial for successful colonisation. Alkan et al. [39] reported that PacC of *C. gloeosporioides* activates degradative enzymes under alkaline conditions [39]. O’Connell et al. [40] found that *C. higginsianum* and *C. graminicola* contain more genes encoding key enzymes involved in secondary metabolism than *Pyricularia* (*Magnaporthe*) *oryzae* and *Fusarium graminearum*. Some of these genes are essential for maintaining primary and secondary metabolism and encode mycotoxins that are important for pathogenicity [40].

The nature of infection by *Colletotrichum* species through the formation of appressoria and penetration of the cuticle and epidermal cells is important in the design of bioassays to determine pathogenicity. A bioassay to assess the ability of *Colletotrichum* isolates to cause infection must not be based solely on wounding the cuticle and epidermis before placing the inoculum over the wound site.

## 3. Citrus Diseases Associated with *Colletotrichum* Species

Globally, citrus diseases caused by *Colletotrichum* species have been reported from every continent. Anthracnose, twig and shoot dieback or withertip, post-bloom fruit drop (PFD), and Key lime anthracnose are important citrus diseases associated with *Colletotrichum* species [41,42,43,44,45].

### 3.1. Anthracnose

Anthracnose is the most typical citrus disease associated with *Colletotrichum* species and affects citrus both pre- and post-harvest. To date, anthracnose has been reported globally from *Citrus* × *aurantium*, *Citrus* × *latifolia*, *Citrus × paradisi*, *Citrus* × *sinensis*, *Citrus aurantiifolia*, *Citrus australasica*, *Citrus bergamia*, *Citrus digitata*, *Citrus flamea*, *Citrus floridana*, *Citrus grandis*, *Citrus hystrix*, *Citrus japonica*, *Citrus limon*, *Citrus maxima*, *Citrus medica*, *Citrus nobilis*, *Citrus reticulata*, *Citrus nobilis* × *Citrus deliciosa*, and the mandarin-like hybrid ‘Mandared’ (Table 1).

As a pre-harvest disease of citrus, *Colletotrichum* infects leaves, stems, flower petals, and unharvested fruit, with notable anthracnose symptoms including sunken necrotic lesions and concentric rings of conidial masses [46,47]. Symptoms on leaves include necrotic lesions, greasy spots, mesophyll collapse, or blight, leading to wilting, yellowing, and leaf drop [42,48,49,50,51,52,53]. Necrotic spots, canker, or splitting are common symptoms on stems, branches, and petioles [45,49]. Fruits infected by *Colletotrichum* often develop sunken, brown or black tear stains, spots, or lesions on the rind. Yellowing of immature fruit, rind russeting and microcracking, and fruit dryness have also been reported as anthracnose symptoms on citrus fruit [42,48,49,52,53,54,55,56,57]. Stem end rot, in which the disease progresses downwards from infected twigs to the stem end of the fruit, is a severe pre-harvest citrus anthracnose disease. *Colletotrichum* spp. cause circular, brown, soft to semi-pliable lesions at the stem end of the fruit, leading to fruit rot, fruit drop, and fruit mummification. Infection has also been detected at both the stem and stylar ends of the fruit [44,45].

Post-harvest anthracnose refers to anthracnose that develops during post-harvest storage, transport, or marketing. *Colletotrichum* species remain quiescent after entering immature citrus fruit and cause anthracnose when the fruit starts to ripen, especially during the post-harvest period [2,12]. The fruit becomes more suitable for disease development because of physiological maturation and modifications induced by effectors produced by *Colletotrichum* species, including a pH range of 5.8 to 6.5, rind softening, and increased soluble carbohydrate content [37,38,39,40]. Sunken, black spots and tear stains are common early-stage symptoms, and the lesions continue to expand and eventually become firm and dry as the fruit becomes overmature or is exposed to stress. Severely infected fruit becomes soft, and orange conidial masses appear at the centre of the lesions [36].

The economic impact caused by anthracnose is profound. Citrus fruit losses can be as high as 90–100% when environmental conditions are suitable for infection [58]. Severe pre-harvest anthracnose symptoms on orange fruit were observed from 2010 to 2013 in Italy [59]. Cheng et al. (2013) reported that leaf drop and fruit spots caused an average yield loss of 15% in *C. reticulata* cv. Shiyue Ju in China in 2008 [60]. Fruit drop caused by stem end rot led to significant economic losses in Kinnow mandarin production by negatively affecting fruit yield and quality [44]. For post-harvest anthracnose, an estimated infection incidence of 15% was observed in grapefruit in Mexico during June 2019 [61]; approximately 10% of orange fruit were infected in China in July 2017 and 2018 [62]; and about 15% of Key lime fruit were infected in China in 2016 [63].

### 3.2. Twig and Shoot Dieback or Withertip

Twig and shoot dieback, which is also known as citrus withertip, has attracted increasing attention in recent years [51,64]. Twig dieback has been frequently reported from multiple commercial citrus species and varieties, including *Citrus* × *aurantium*, *Citrus* × *latifolia*, *Citrus margarita* (syn. *Fortunella margarita*), *Citrus* × *sinensis*, *Citrus* × *tangelo*, *Citrus trifoliata* (syn. *Poncirus trifoliata*), *Citrus aurantiifolia*, *Citrus limon*, *Citrus maxima*, *Citrus medica*, *Citrus × paradisi*, *Citrus reticulata* and the mandarin-like hybrid ‘Mandared’ (Table 1).

Typical symptoms of twig dieback include gumming on twigs, twig blight, twig and branch dieback, crown thinning, leaf chlorosis, leaf necrosis, and defoliation [42,46,51,64,65]. The disease starts from the tips of the twigs, spreads towards the main stem, and eventually infects the whole plant, negatively affecting normal plant growth, structure, and function, and potentially leading to plant death [46,66,67]. An outbreak of severe twig dieback resulted in a 40% yield loss in *Citrus reticulata* cv. Kinnow in Pakistan in 2014 [65]. Moreover, twig dieback caused by *Colletotrichum* spp. is likely to be a source of inoculum that spreads to flower petals and then to young fruit, leading to post-harvest anthracnose [46,53]. The pathogen can survive in leaves and twigs for a long period before flowers are present [68]. Camiletti et al. (2022) reported latent infection of *Colletotrichum* dieback on citrus leaves and twigs in California [64].

### 3.3. Post-Bloom Fruit Drop

Post-bloom fruit drop (PFD), or blossom blight, is another important citrus disease caused by *Colletotrichum* species. It was initially reported in Belize and is now widely distributed in tropical and subtropical regions of the Americas [69,70,71,72,73,74,75]. *Colletotrichum* species cause PFD in all commercial citrus varieties, while sweet orange (*Citrus* × *sinensis*) is the most common host species [69,73,75,76].

Post-bloom fruit drop develops quickly under highly humid conditions during the bloom period, and disease symptoms appear within 48 to 72 h after initial infection [75]. Peach-to-brown, water soaked, elongated lesions first appear on the petals and filaments of diseased flowers. The lesions continuously expand, causing blight of the whole flower and eventually infecting the entire flower cluster. As the disease develops, the pathogen enters the fruit, resulting in yellowing and abscission of the developing fruit [74,75]. Persistent calyces remaining attached to the peduncle are an important and specific symptom for diagnosing PFD [42,75,76]. The observation of petal lesions alone on citrus plants is considered pre-harvest anthracnose [48,52].

Severe outbreaks of PFD cause extensive losses of immature citrus fruit. Fruit losses of sweet orange, grapefruit, lemon and Tahiti lime ranged from 25 to 35% in Bermuda [75]. In Brazil, PFD may cause severe yield losses of up to 93% when the bloom period coincides with rainy conditions [76].

### 3.4. Key Lime Anthracnose

Key lime anthracnose is a specific disease complex affecting only the leaves, twigs, flowers and fruit of Key lime [42,70,77]. Common symptoms include necrotic lesions on leaves, twigs, flowers, and fruit, ranging from small lesions to necrotic blight of entire shoots and inflorescences [71,78]. The disease has been observed in citrus orchards in Belize, Brazil, Costa Rica, Mexico, Panama, the Dominican Republic, and the USA, and has been suggested to be limited to the Americas [48,71,78].

## 4. Global Diversity and Distribution of Citrus-Associated *Colletotrichum* Species

Thirty-nine *Colletotrichum* species have been isolated from a range of *Citrus* species globally. Most *Colletotrichum* species have been reported as plant pathogens, while several species have been found to exhibit endophytic or latent lifestyles, or have been observed as saprobes (Table 1). In *Colletotrichum*, a monophyletic clade formed by a group of species exhibiting similar morphological characteristics is defined as a species complex, or aggregate [5,79].

### 4.1. Gloeosporioides Complex

The gloeosporioides complex is the most commonly reported species complex associated with citrus. Sixteen species belonging to the gloeosporioides complex have been reported as pathogens, endophytes, or saprobes on citrus (Table 1).

*Colletotrichum gloeosporioides* is the most common *Colletotrichum* species associated with citrus globally [42,43,46]. The species was originally isolated from orange (*Citrus* × *sinensis*) in southern Italy and has been found to be the most prevalent pathogen causing pre- and post-harvest anthracnose of multiple *Citrus* species and varieties in Australia, China, and Europe [5,42,49,54]. Previously, *C. gloeosporioides* was reported to cause leaf anthracnose in Australia, China, Greece, Indonesia, Italy, Mexico, Portugal, Spain, Thailand, Tunisia, and Turkey [42,45,48,49,50,51,52,53,57,59,80,81,82]; flower anthracnose in Portugal, Spain, and Tunisia [42,48,52]; branch anthracnose in Portugal [48]; stem spot in Australia [49]; and pre- and post-harvest fruit anthracnose in Australia, Brazil, China, Ghana, Greece, Indonesia, Italy, Mexico, Morocco, Portugal, South Africa, Thailand, Tunisia, Turkey, Vietnam, and Zimbabwe [42,45,48,49,52,53,54,55,56,57,59,61,63,80,81,82,83]. *Colletotrichum gloeosporioides* has also been reported as the most prevalent species causing citrus twig dieback in Australia and Italy and has been associated with citrus twig dieback or withertip in Algeria, China, Greece, Malta, Morocco, Pakistan, Portugal, Spain, Thailand, Tunisia, Turkey, and the USA [42,45,46,51,52,53,54,56,58,67,80,81,84,85,86]. In Brazil, Bermuda, and Mexico, *C. gloeosporioides* was found to cause post-bloom fruit drop of *Citrus limon* and *Citrus* × *sinensis* [57,69,73,75,76]. Moreover, *C. gloeosporioides* has also been found to exhibit an endophytic lifestyle in asymptomatic branches and leaves in China and latent infection in asymptomatic tissues in the USA [43,64,87]. Saprobic *C. gloeosporioides* has been found on dead fallen leaves in Australia, Malta and China [42,43,49].

*Colletotrichum siamense* is the second most important species in the gloeosporioides complex. The species has been reported to cause citrus fruit anthracnose in Australia, Bangladesh, China, Egypt, India, Thailand, and Vietnam [44,45,49,54,60,80,88]; leaf anthracnose in China and Thailand [45,60,80,85]; branch canker in Thailand [45]; and twig dieback or withertip in Pakistan and Thailand [45,65,67,80]. *Colletotrichum siamense* has also been reported as an endophyte isolated from asymptomatic leaves in China [87,89].

*Colletotrichum fructicola* is the third most common species in the gloeosporioides complex. Globally, *C. fructicola* has been found to infect citrus leaves in China and Thailand [45,50,80], branches in Australia [49], twigs in Tunisia [53], and fruit in Australia and China [49,62]. Endophytic *C. fructicola* has also been isolated from asymptomatic leaves in China [43,87].

In addition, *C. australianum* was associated with fruit anthracnose in Australia [49]. *Colletotrichum endophyticum* was found to cause leaf spot in Thailand, as well as fruit and leaf anthracnose in Indonesia [45,82]. *Colletotrichum helleniense* was found to infect twigs and fruit in Greece [42]. *Colletotrichum hystricis* and *C. cigarro* were reported to cause leaf disease in Italy [42,90]. *Colletotrichum queenslandicum* was associated with fruit anthracnose in Indonesia and leaf anthracnose in Indonesia and the USA [82,91]. *Colletotrichum syzygicola* was reported to cause fruit anthracnose in Thailand [92]. *Colletotrichum theobromicola* was associated with fruit anthracnose in Australia [49]. *Colletotrichum aenigma* was isolated from *Citrus* × *sinensis* in Italy [93]. An unidentified *Colletotrichum* species was found to infect citrus leaves and fruit in Australia [49]. *Colletotrichum tainanense* and *C. tomentosae* were found as endophytes in asymptomatic leaves in China, while *C. asianum* was found as an endophyte in asymptomatic twigs in China [89].

### 4.2. Boninense Complex

The boninense complex is the second most commonly reported species complex associated with citrus globally. Seven species belonging to the boninense complex have been reported as pathogens, endophytes, or saprobes on citrus (Table 1).

*Colletotrichum karstii* is the most important species in the boninense complex and the second most prevalent *Colletotrichum* pathogen associated with citrus globally. *Colletotrichum karstii* has frequently been found to cause pre- and post-harvest anthracnose on multiple *Citrus* species and varieties across a wide geographical distribution, including leaf anthracnose in Australia, China, Italy, Portugal, Spain, and Turkey [42,43,48,49,50,59,81]; branch anthracnose in Portugal [48]; and pre- and post-harvest fruit anthracnose in Italy, Portugal, South Africa, Tunisia, and Turkey [42,48,52,54,59,81,94]. *Colletotrichum karstii* has also been associated with citrus twig dieback or withertip in Australia, Italy, Malta, Portugal, Spain, Turkey, and the USA [42,51,81,85,86,94]. Moreover, *C. karstii* has been reported to exhibit an endophytic lifestyle in asymptomatic branches, leaves and shoots in China, as well as latent infection in asymptomatic tissues in the USA [43,64,87].

*Colletotrichum novae-zelandiae* is the second most important species in the boninense complex. The species has been reported to cause citrus twig dieback or withertip in Australia and Malta [42,86], leaf lesions in Greece and Malta [42], and fruit infection in New Zealand [95].

*Colletotrichum catinaense* is the third most common species in the boninense complex and has been found to cause twig withertip and leaf lesions in Italy, as well as fruit tear stain in Portugal [42].

In addition, *C. boninense* has been reported to cause leaf anthracnose in China [50], *C. constrictum* has been associated with fruit rot in New Zealand [95], and *C. limonicola* has been found to cause leaf lesions in Malta [42]. *Colletotrichum citricola* has been reported to exhibit an endophytic lifestyle in asymptomatic leaves and has been reported as a saprobe on leaves in China [43,87].

### 4.3. Acutatum Complex

The acutatum complex is the third most commonly reported species complex associated with citrus. Seven species belonging to the acutatum complex have been reported as pathogens on citrus (Table 1).

*Colletotrichum abscissum* has been reported as the causal agent of post-bloom fruit drop in Brazil and the USA [69,72,73,74,75,76]. This species was previously identified as *C. acutatum* and was therefore recorded as being associated with post-bloom fruit drop under that name [75,76]. It was later reclassified as *C. abscissum* [72].

*Colletotrichum limetticola* has been found to cause Key lime anthracnose in the USA [70]. This species was initially identified as *C. acutatum* and was therefore recorded as being associated with Key lime anthracnose under that name [75,78]. It was later reclassified as *C. limetticola* [70].

In addition, *C. johnstonii* has been associated with citrus fruit rot in Australia and New Zealand [70,96], *C. acutatum* has been reported to cause leaf lesions in Italy [42], *C. godetiae* has been found to infect the fruit of *Citrus × aurantium* [70], *C. citri* has been reported to cause shoot anthracnose in China [43], and *C. simmondsii* has been associated with fruit anthracnose in China [9].

### 4.4. Magnum Complex

Three species belonging to the magnum complex have been reported as pathogens or endophytes on citrus (Table 1). *Colletotrichum brevisporum* has been found to cause leaf anthracnose and to exhibit an endophytic lifestyle in China [5,50]. *Colletotrichum guangdongense* has been found as an endophyte in asymptomatic twigs in China [89]. *Colletotrichum kokhaense* has been associated with leaf lesions in Thailand [80].

### 4.5. Gigasporum Complex

Two species belonging to the gigasporum complex have been reported as pathogens or endophytes on citrus (Table 1). *Colletotrichum gigasporum* has been reported to cause twig dieback and leaf lesions in Thailand [80]. The species has also been found as an endophyte in asymptomatic leaves in China [87]. *Colletotrichum citri-maximae* has been found to exhibit an endophytic lifestyle in asymptomatic fruit in China [54].

### 4.6. Dracaenophilum, Orchidearum and Truncatum Complexes

*Colletotrichum tropicicola*, belonging to the dracaenophilum complex, has been reported to cause leaf lesions and twig dieback in Thailand [80,97]. Endophytic *C. tropicicola* has also been isolated from asymptomatic leaves in China [87]. *Colletotrichum tropicicola* has also been isolated from *Citrus* sp. in Mexico [98].

*Colletotrichum plurivorum*, belonging to the orchidearum complex, has been reported to infect citrus leaves in Thailand [45,80]. The species has also been found as an endophyte in asymptomatic leaves in China [87,89]. *Colletotrichum truncatum*, belonging to the truncatum complex, has been found to cause shoot and leaf anthracnose and to exhibit an endophytic lifestyle in asymptomatic leaves in China [43,87].

### 4.7. Singleton Species

The singleton species *C. citrus-medicae* has been reported to cause leaf spot of *Citrus medica* in China [99].

These identified citrus-associated *Colletotrichum* species have important implications for plant biosecurity risk management and the development of integrated control strategies. However, some of these *Colletotrichum* species may have been misidentified by analysing only morphological characters [56], only ITS sequences [52,53,59,84], or species-specific primers designed before the adoption of multi-locus phylogeny [75,76]. Phylogenetic analyses based on the combination of ITS and *gapdh* sequences [55] or ITS and *tub2* sequences [93] are also unable to clearly distinguish a *Colletotrichum* species from closely related species [5]. Further identification of *Colletotrichum* species should utilise the polyphasic approach, which consists of multi-gene phylogenetic analysis, traditional morphological character descriptions, and other information such as geographical and ecological data.

**Table 1:** *Colletotrichum* species associated with *Citrus*, with detailed information on *Citrus* species, host organ, disease symptom, country and references.

*Colletotrichum* Species	Host Species	Host Organ	Disease Symptom	Country	Reference
**Gloeosporioides complex**					
***C. aenigma***	*Citrus* × *sinensis*	NA	NA	Italy	[93]
***C. asianum***	*Citrus grandis*	Asymptomatic twig	Endophyte	China	[89]
***C. australianum***	*Citrus reticulata*	Fruit	Anthracnose	Australia	[49]
	*Citrus* × *sinensis*	Fruit	Anthracnose	Australia	[49]
***C. endophyticum***	*Citrus aurantiifolia*	Leaf	Leaf spot	Thailand	[45]
	*Citrus reticulata*	Fruit or leaf	Anthracnose	Indonesia	[82]
***C. fructicola***	*Citrus aurantiifolia*	Leaf	Leaf spot	Thailand	[45,80]
	*Citrus bergamia*	Leaf	Anthracnose	China	[50]
	*Citrus grandis*	Leaf	Anthracnose	China	[50]
	*Citrus × latifolia*	Branch	Splitting	Australia	[49]
	*Citrus maxima*	Leaf	Leaf spot	Thailand	[45,80]
	*Citrus maxima*	Asymptomatic leaf	Endophyte	China	[87]
	*Citrus nobilis*	Asymptomatic leaf	Endophyte	China	[87]
	*Citrus reticulata*	Fruit	Anthracnose	Australia	[49]
	*Citrus reticulata*	Asymptomatic leaf	Endophyte	China	[43,87]
	*Citrus* × *sinensis*	Fruit	Anthracnose	China	[62]
	*Citrus* × *sinensis*	Twig	Anthracnose	Tunisia	[53]
	*Citrus tachibana*	Asymptomatic leaf	Endophyte	China	[87]
***C. gloeosporioides***	*Citrus aurantiifolia*	Fruit	Anthracnose, Dryness, Fruit Rot	Australia, China	[49,63]
		Leaf	Leaf spot, Leaf lesion	Thailand	[45,80]
		Twig	Twig dieback	Thailand	[80]
		Fruit or leaf	Anthracnose	Indonesia	[82]
	*Citrus* × *aurantium*	Leaf	Anthracnose	Portugal	[48]
		Twig	Twig dieback	Thailand	[80]
	*Citrus bergamia*	Fruit	Fruit lesion	Greece	[42]
	*Citrus digitata*	Leaf	Leaf lesion	Italy	[42]
	*Citrus floridana*	Fruit	Fruit lesion	Italy	[42]
	*Citrus grandis*	Leaf	Anthracnose	China	[50]
		Asymptomatic branch	Endophyte	China	[43]
		Asymptomatic leaf	Endophyte	China	[43]
	*Citrus japonica*	Fruit	Anthracnose	Australia	[49]
	*Citrus limon*	Branch	Anthracnose	Portugal	[48]
		Flower	Anthracnose	Portugal	[48]
		Calyx	Persistent calyx	Mexico	[57]
		Immature fruit	Yellowish immature fruits	Mexico	[57]
		Fruit	Fruit lesion, Anthracnose	Mexico, Portugal, Turkey, Vietnam	[48,54,81]
		Leaf	Leaf lesion, Anthracnose	Australia, China, Mexico, Portugal, Spain	[42,48,49,50,57]
		Stem	Stem spot	Australia	[49]
		Twig	Twigs withertip	Algeria, Italy, Malta, Turkey	[42,81,84]
		Fruit or leaf	Anthracnose	Indonesia	[82]
		Dead fallen leaf	Saprobe	Australia	[49]
	*Citrus maxima*	Leaf	Leaf lesion	Thailand	[80]
		Twigs	Twigs withertip	Greece	[42]
		Asymptomatic leaf	Endophyte	China	[87]
	*Citrus medica*	Leaf	Leaf lesion	Italy	[42]
	*Citrus nobilis*	Fruit	Anthracnose	Vietnam	[83]
		Asymptomatic leaf	Endophyte	China	[87]
	*Citrus × paradisi*	Fruit	Anthracnose	Mexico	[61]
		Leaf	Leaf lesion	Portugal, Spain	[42]
		Twig	Twigs withertip	Italy, Spain	[42]
	*Citrus reticulata*	Flower	Anthracnose	Portugal	[48]
		Fruit	Russeting, microcracking, Fruit spot, Fruit lesion, Anthracnose	Australia, Portugal, Thailand, Turkey	[45,48,49,80,81]
		Leaf	Leaf lesion, Leaf spot	Australia, China, Greece, Italy, Thailand, Turkey	[42,49,50,80,81]
		Twig, shoot	Twig dieback, Twigs withertip, Twig blight	Australia, Italy, Spain, Thailand, Tunisia, Turkey, USA	[42,45,46,51,80,81,85,86]
		Fruit or leaf	Anthracnose	Indonesia	[82]
		Asymptomatic branch	Endophyte	China	[43]
		Asymptomatic leaf	Endophyte	China	[43,87]
		Asymptomatic tissue	Latent infection	USA	[64]
	*Citrus* × *sinensis*	Branch	Anthracnose	Portugal	[48]
		Flower	Post-bloom fruit drop	Brazil, Bermuda	[69,73,75,76]
		Flower	Petal lesions	Spain, Tunisia	[42,52]
		Flower	Anthracnose	Portugal	[48]
		Fruit	Fruit lesion, Fruit tear stain, Anthracnose	Australia, Brazil, Ghana, Italy, Morocco, Portugal, South Africa, Tunisia, Zimbabwe	[42,48,49,52,53,54,55,56]
		Leaf	Leaf lesion, Leaf greasy spot, necrotic patches and mesophyll collapse, Anthracnose	Australia, China, Italy, Portugal, Spain, Tunisia	[42,48,49,50,51,52,53]
		Stem	Stem spot	Australia	[49]
		Twig, shoot	Twig dieback, Twigs withertip	Australia, Algeria, Italy, Morocco, Portugal, Tunisia, Turkey, USA	[42,51,52,53,56,81,84,85,86]
		Preharvest fruits or leaves	Anthracnose	Italy	[59]
		Asymptomatic leaf	Endophyte	China	[43]
		Leaf litter	Saprobe	Malta	[42]
		Asymptomatic tissue	Latent infection	USA	[64]
	*Citrus tachibana*	Asymptomatic leaf	Endophyte	China	[87]
	*Citrus* × *tangelo*	Twig	Twig dieback, Twigs withertip	Tunisia	[46]
	*Citrus unchiu*	Asymptomatic branch	Endophyte	China	[43]
		Asymptomatic leaf	Endophyte	China	[43]
		Leaf	Saprobe	China	[43]
	*Citrus* sp.	Twig	Twigs withertip	China, Pakistan	[58,67]
	Mandarin-like hybrid ‘Mandared’	Twig	Twig dieback	Italy	[51]
		Leaf	Necrotic patches and mesophyll collapse	Italy	[51]
***C. helleniense***	*Citrus reticulata*	Fruit	Fruit lesion	Greece	[42]
	*Citrus trifoliata* (syn. *Poncirus trifoliata*)	Twig	Twigs withertip	Greece	[42]
***C. hystricis***	*Citrus hystrix*	Leaf	Leaf lesion	Italy	[42]
***C. cigarro***	*Citrus reticulata*	Leaf	Light brown necrotic lesions	Italy	[90]
***C. queenslandicum***	*Citrus aurantiifolia*	Fruit or leaf	Anthracnose	Indonesia	[82]
	*Citrus* × *latifolia*	Leaf	Anthracnose	USA	[91]
***C. siamense***	*Citrus australasica*	Fruit	Anthracnose	Australia	[49]
	*Citrus aurantiifolia*	Fruit	Anthracnose	Thailand	[80]
		Leaf	Leaf spot	Thailand	[45,80]
		Twig	Twig dieback	Thailand	[80]
	*Citrus* × *aurantium*	Twig	Twig dieback	Thailand	[80]
		Leaf	Leaf lesion	Thailand	[80]
	*Citrus grandis*	Asymptomatic leaf	Endophyte	China	[89]
	*Citrus hystrix*	Fruit	Fruit stem end rot	Thailand	[45]
	*Citrus* × *latifolia*	Twig	Twig dieback	Thailand	[80]
	*Citrus limon*	Fruit	Anthracnose	Australia	[49]
		Fruit	NA	Vietnam	[54]
	*Citrus maxima*	Branch	Branch canker	Thailand	[45]
		Leaf	Leaf spot	Thailand	[80,85]
		Asymptomatic leaf	Endophyte	China	[87]
	*Citrus nobilis*	Asymptomatic leaf	Endophyte	China	[87]
	*Citrus nobilis* × *Citrus deliciosa*	Fruit	Fruit stem end rot, fruit drop	India	[44]
		Twig	Twig drying	India	[44]
	*Citrus pennivesiculata*	Fruit	NA	Bangladesh, Egypt	[54]
	*Citrus reticulata*	Fruit	Fruit spot	Thailand, China	[45,60]
		Leaf	Leaf spot	China, Thailand	[45,60]
		Twig	Twig dieback, Twigs withertip	Pakistan, Thailand	[45,65]
	*Citrus* × *sinensis*	Fruit	Fruit Rot	India	[88]
	*Citrus* sp.	Twig	Twigs withertip	Pakistan	[67]
***C. syzygicola***	*Citrus aurantiifolia*	Fruit	Anthracnose	Thailand	[92]
***C. tainanense***	*Citrus grandis*	Asymptomatic leaf	Endophyte	China	[89]
***C. theobromicola***	*Citrus aurantiifolia*	Fruit	Anthracnose	Australia	[49]
***C. tomentosae***	*Citrus grandis*	Asymptomatic leaf	Endophyte	China	[89]
***Colletotrichum*** **sp.**	*Citrus australasica*	Leaf/fruit	spot	Australia	[49]
**Boninense complex**					
***C. boninense***	*Citrus medica*	Leaf	Anthracnose	China	[50]
***C. catinaense***	*Citrus aurantiifolia*	Twigs	Twigs withertip	Italy	[42]
	*Citrus reticulata*	Leaf	Leaf lesion	Italy	[42]
	*Citrus* × *sinensis*	Fruit	Fruit tear stain	Portugal	[42]
***C. citricola***	*Citrus maxima*	Asymptomatic leaf	Endophyte	China	[87]
	*Citrus unchiu*	Leaf	Saprobe	China	[43]
***C. constrictum***	*Citrus limon*	Fruit	Fruit rot	New Zealand	[95]
***C. karstii***	*Citrus grandis*	Asymptomatic leaf	Endophyte	China	[43]
		Asymptomatic shoot	Endophyte	China	[43]
		Asymptomatic branch	Endophyte	China	[43]
	*Citrus limon*	Branch	Anthracnose	Portugal	[48]
		Fruit	Fruit lesion, Anthracnose	Italy, Portugal, South Africa, Turkey	[42,48,54,81,94]
		Leaf	Leaf lesion, Anthracnose	Australia, China, Portugal, Spain, Turkey	[42,43,48,49,81]
		Twigs	Twigs withertip	Portugal, Turkey	[42,94]
		Asymptomatic branch	Endophyte	China	[43]
	*Citrus margarita* (syn. *Fortunella margarita*)	Leaf	Leaf lesion	Italy	[42]
		Fruit	Fruit tear stain	Italy	[42]
		Twig	Twig dieback	Italy	[100]
		Branch	Branch dieback	Italy	[100]
	*Citrus maxima*	Asymptomatic leaf	Endophyte	China	[87]
	*Citrus nobilis*	Asymptomatic leaf	Endophyte	China	[87]
	*Citrus × paradisi*	Leaf	Leaf lesion	Spain	[42]
		Twigs	Twigs withertip	Italy	[42]
	*Citrus reticulata*	Leaf	Leaf lesion	China, Italy	[42,50]
		Twig, shoot	Twig dieback, Twigs withertip	Australia, Spain, Turkey, USA	[42,81,85,86]
		Asymptomatic leaf	Endophyte	China	[87]
		Asymptomatic tissue	Latent infection	USA	[64]
	*Citrus* × *sinensis*	Fruit	Fruit lesion	Italy, South Africa, Tunisia	[42,52,54]
		Leaf	Leaf lesion, Leaf greasy spot	Australia, China, Italy, Spain	[42,49,50]
		Twig, shoot	Twig dieback, Twigs withertip	Australia, Italy, Malta, Portugal, USA	[42,51,85,86]
		Asymptomatic tissue	Latent infection	USA	[64]
		Preharvest fruits or leaves	Anthracnose	Italy	[59]
	*Citrus tachibana*	Asymptomatic leaf	Endophyte	China	[87]
	*Citrus* sp.	NA	NA	New Zealand	[95]
***C. limonicola***	*Citrus limon*	Leaf	Leaf lesion	Malta	[42]
***C. novae-zelandiae***	*Citrus limon*	Twigs	Twigs withertip	Malta	[42]
		Leaf	Leaf lesion	Malta	[42]
	*Citrus × paradisi*	Fruit	NA	New Zealand	[95]
		Leaf	Leaf lesion	Greece	[42]
	*Citrus reticulata*	Twig	Twig dieback	Australia	[86]
	*Citrus* × *sinensis*	Twig	Twig dieback, Twigs withertip	Australia, Malta	[42,86]
**Acutatum complex**					
***C. abscissum***	*Citrus* × *sinensis*	Flowers	Post-bloom fruit drop	Brazil, USA	[69,72,73,74,75,76]
***C. acutatum***	*Citrus limon*	Leaf	Leaf lesion	Italy	[42]
	*Citrus* × *sinensis*	Leaf	Leaf lesion	Italy	[42]
***C. citri***	*Citrus aurantiifolia*	Shoot	Anthracnose	China	[43]
***C. godetiae***	*Citrus × aurantium*	Fruit	Fruit rot	Unknown	[70]
***C. johnstonii***	*Citrus australasica*	Fruit	Fruit rot	Australia	[96]
	*Citrus* sp.	Fruit	Fruit rot	New Zealand	[70]
***C. limetticola***	Key lime (*Citrus aurantiifolia*)	Twig	Key lime anthracnose	USA	[70]
***C. simmondsii***	*Citrus reticulata*	Fruit	Anthracnose	China	[9]
**Magnum complex**					
***C. brevisporum***	*Citrus grandis*	NA	Endophyte	China	[5]
	*Citrus medica*	Leaf	Anthracnose	China	[50]
***C. guangdongense***	*Citrus grandis*	Asymptomatic twig	Endophyte	China	[89]
***C. kokhaense***	*Citrus aurantiifolia*	Leaf	Leaf lesion	Thailand	[80]
**Gigasporum complex**					
***C. citri-maximae***	*Citrus maximum*	Asymptomatic fruit	Endophyte	China	[54]
***C. gigasporum***	*Citrus aurantiifolia*	Twig	Twig dieback	Thailand	[80]
	*Citrus maxima*	Leaf	Leaf lesion	Thailand	[80]
	*Citrus nobilis*	Asymptomatic leaf	Endophyte	China	[87]
**Dracaenophilum complex**					
***C. tropicicola***	*Citrus maxima*	Leaf	Leaf lesion	Thailand	[80,97]
		Twig	Twig dieback	Thailand	[80]
		Asymptomatic leaf	Endophyte	China	[87]
	*Citrus* × *latifolia*	Leaf	Leaf lesion	Thailand	[80]
	*Citrus* sp.	NA	NA	Mexico	[98]
**Orchidearum complex**					
***C. plurivorum***	*Citrus aurantiifolia*	Leaf	Leaf spot	Thailand	[45,80]
	*Citrus grandis*	Asymptomatic leaf	Endophyte	China	[89]
	*Citrus maxima*	Leaf	Leaf lesion	Thailand	[80]
	*Citrus nobilis*	Asymptomatic leaf	Endophyte	China	[87]
	*Citrus tachibana*	Asymptomatic leaf	Endophyte	China	[87]
**Truncatum complex**					
***C. truncatum***	*Citrus flamea*	Shoot	Anthracnose	China	[43]
	*Citrus limon*	Leaf	Anthracnose	China	[43]
	*Citrus tachibana*	Asymptomatic leaf	Endophyte	China	[87]
**Singleton species**					
***C. citrus-medicae***	*Citrus medica*	Leaf	Leaf spot	China	[99]

## 5. Pathogenicity and Host- or Tissue-Specificity of Citrus-Associated *Colletotrichum* Species

Pathogenicity, which indicates the ability of a pathogen to cause disease on a host, encompasses variations in aggressiveness and pathotype differences. Different *Colletotrichum* species may contain isolates with various levels of aggressiveness and different pathotypes on the same host species [42,45]. Different pathotypes within the same *Colletotrichum* species can be identified on the same host species according to qualitative differences in disease expression [101,102].

Controlled environment bioassays using detached or *in planta* leaves, twigs, flower petals, and fruit are widely used to test the pathogenicity of *Colletotrichum* species. Many pathogenicity tests have been performed on *Citrus* by inoculating *Colletotrichum* isolates onto the same tissues of the same host species to reproduce similar disease symptoms and then re-isolating the pathogens from the inoculated tissues [88,96]. Studies have reported that *Colletotrichum* isolates obtained from one host or tissue may also cause symptoms on other hosts or tissues, suggesting that these species are not strictly host- or tissue-specific. However, these findings are often dependent on the type of bioassay. Cross-host inoculation bioassays have been conducted to test the pathogenicity of *Colletotrichum* species associated with citrus disease on multiple hosts and tissues, and several species have demonstrated the ability to infect citrus hosts or tissues beyond the original host tissue using wound or non-wound inoculation (Table 2).

Similarly, *Colletotrichum* species have been shown to infect and cause disease across plant genera. *Colletotrichum acutatum* isolated from diseased fruit of olive (*Olea europaea*) and *C. karstii* isolated from diseased leaves of camellia (*Camellia* sp.) caused disease symptoms on wounded *in planta* twigs of sweet orange ‘Tarocco Scirè’, lemon ‘Femminello 2Kr’, and bergamot ‘Fantastico’; wounded detached leaves of sweet orange ‘Moro’ and ‘Navelina’; and wounded fruit of apple (*Malus domestica*) ‘Fuji’ and ‘Cripps Pink’. The *C. acutatum* and *C. karstii* isolates caused significantly larger lesion areas on wounded leaves of sweet orange ‘Navelina’ than those caused by *Colletotrichum* isolates associated with citrus disease [51]. *Colletotrichum gloeosporioides* isolated from diseased mandarin and sweet orange twigs, diseased sweet orange leaves, and diseased lemon fruit, as well as *C. karstii* isolated from diseased sweet orange twigs, infected wounded fruit of apple ‘Fuji’ and ‘Cripps Pink’ [51]. *Colletotrichum gloeosporioides* associated with lemon twig withertip caused lesions on wounded fruit of apple ‘Gala’, avocado ‘Fuerte’, pear ‘Santa Maria’, pepper ‘Capia’, and banana ‘Cavendish’ [81]. *Colletotrichum karstii* causing kumquat twig and branch dieback infected wounded fruit of apple ‘Golden Delicious’ [100]. These results may however overstate the seriousness of cross-host pathogenicity because the bioassays involved wounding the cuticle and epidermis of the plant tissue with a sterile needle or scalpel before placing the inoculum over the wound.

The cuticle and epidermis are important physical barriers that defend the host against pathogen infection. Artificial damage to these physical barriers bypasses the need for *Colletotrichum* species to penetrate the cuticle and epidermis and may therefore provide a biassed measure of pathogenicity [27,28,101,103]. In contrast, during non-wound inoculation, the inoculum is placed directly on the surface of the host tissue, requiring the *Colletotrichum* species to penetrate the physical barriers to initiate infection. This method is closer to infection occurring in the natural environment [2,102,103]. Hence, the non-wound inoculation method tends to provide a more realistic evaluation of the pathogenicity of weakly aggressive species or species with latent or endophytic lifestyles [101,102]. *Colletotrichum gigasporum*, *C. kokhaense*, *C. gloeosporioides*, *C. plurivorum*, and *C. siamense* showed various degrees of aggressiveness on non-wounded lime and orange fruit but showed similarly high levels of aggressiveness when the citrus fruit was wounded before inoculation [80]. *Colletotrichum gloeosporioides*, *C. karstii*, and *C. novae-zelandiae*, which developed a latent lifestyle after infecting non-wounded orange and lemon twigs, became pathogenic when the citrus twigs were inoculated using the wound inoculation method [86].

## 6. Biosecurity Implications of Citrus-Associated *Colletotrichum* Species

Thirty-nine *Colletotrichum* species have been found to be associated with *Citrus* species, with additional novel species continuing to be described [1,5]. Most *Colletotrichum* species have been collected from a range of *Citrus* species and host tissues, except for species represented by only one isolate. Host- or tissue-specific pathogens typically have a more restricted distribution, whereas those with a broad host range are more likely to cause disease worldwide. *Colletotrichum gloeosporioides*, which is the most important *Colletotrichum* species associated with citrus globally, has also been reported from the greatest diversity of *Citrus* species and can infect all citrus tissues [42,43,46,73]. Isolates of many citrus-associated *Colletotrichum* species, such as *C. fructicola*, *C. gigasporum*, *C. gloeosporioides*, *C. karstii*, *C. plurivorum*, *C. siamense*, *C. theobromicola*, and *C. tropicicola*, have been reported from hosts belonging to different genera [1,104,105,106,107]. Cross-plant genus infection by *Colletotrichum* species has also been demonstrated on wounded fruits [9]. Both *in planta* and detached tissue bioassays demonstrated that *C. acutatum* and *C. karstii* isolates obtained from hosts belonging to different genera were able to infect citrus using wound inoculation [51]. The isolation of the same *Colletotrichum* species from different hosts, together with the first reports of *Colletotrichum* species on citrus in different geographical locations, demonstrates the potential for cross-infection and poses a threat to the biosecurity of the citrus industry.

In addition, all species in the genus *Citrus* are hybrids or introgressions between mandarin (*Citrus reticulata*), pomelo (*Citrus maxima*) and citron (*Citrus medica*) [14,15]. The genetic similarity among commercially cultivated *Citrus* species may be conducive to the transmission of *Colletotrichum* species between different citrus hosts. Meanwhile, the demonstrated cross-host infection by *C. gloeosporioides* isolated from citrus on wounded fruit of apple, avocado, pear, pepper, and banana, and by *C. karstii* isolated from citrus on wounded apple fruit, makes these two species potential high-risk pathogens for different fruit and vegetable industries, especially where susceptible plants are grown near citrus [51,81,100].

An exotic pathogen may rapidly colonise a new environment and pose a biosecurity risk to local plants when environmental conditions are suitable for the species [108]. The temperature tolerance of *Colletotrichum* species is an important factor when considering their distribution and the damage they may cause to the local ecosystem. Although the optimum temperature for mycelial growth, conidial germination, and appressoria formation and melanisation of *C. gloeosporioides* was estimated to be around 24 °C, its growth rate remained relatively high at 30 °C and was only moderately reduced at 35 °C compared with its growth at 25 °C [51,64,76]. The broad temperature tolerance of *C. gloeosporioides* is consistent with its reported occurrence across a wide range of climates, including tropical, subtropical, Mediterranean, temperate, and semi-arid regions (Table 1).

*Colletotrichum siamense* was originally described from Arabica coffee (*Coffea arabica*) in Thailand and has been reported to grow optimally at 30 °C. Consistent with this relatively high optimal growth temperature, previous reports of *C. siamense* from diseased citrus tissues have mainly occurred in tropical climate zones, and *C. siamense* has been identified as the most prevalent *Colletotrichum* species associated with citrus in Thailand [1,80,109]. Furthermore, *C. acutatum* and *C. karstii* have optimum growth temperatures of around 24 °C, and their growth rates were dramatically reduced at 30 and 35 °C, suggesting that these pathogens may be more likely to cause disease on plants in temperate regions [51,64,76]. In contrast, *C. endophyticum*, *C. fructicola*, *C. gigasporum*, *C. plurivorum*, and *C. tropicicola* may cause severe damage to agricultural industries in tropical climate zones [43,45,49,50,80,82,89,97]. Therefore, citrus-associated *Colletotrichum* species may need to be listed as quarantine pathogens by national biosecurity regulatory authorities in countries or states where they have not previously been detected, to reduce potential biosecurity risks to local plants, especially in areas with environmental conditions suitable for these species. Until recently, *C. limetticola* has been designated as a quarantine pest in Morocco. *Colletotrichum acutatum* sensu lato have been regulated as a quarantine pest in Tunisia and Israel, in the A1 list in Chile, Kazakhstan and Armenia, as well as in the A2 list in Serbia and the Eurasian Economic Union (EAEU) [110].

*Colletotrichum* species have multiple lifestyles and can transition to different lifestyle stages during the disease cycle to complete infection [2,11]. Several *Colletotrichum* species associated with citrus diseases, including *C. fructicola*, *C. gloeosporioides*, *C. siamense*, *C. karstii*, *C. brevisporum*, *C. gigasporum*, *C. tropicicola*, *C. plurivorum*, and *C. truncatum*, have been reported as either causing citrus disease or exhibiting endophytic or quiescent lifestyles (Table 1). Although several *Colletotrichum* species have only been observed as endophytes in citrus, their pathogenic potential cannot be excluded. *Colletotrichum gigasporum* and *C. plurivorum* were first identified as endophytes and were later reported to cause citrus disease [45,80,87,89]. Camiletti et al. [64] demonstrated that *C. gloeosporioides* and *C. karstii* isolated from asymptomatic leaves and twigs were able to cause twig dieback. Therefore, the potential dispersal of *Colletotrichum* species in asymptomatic citrus tissues is of major importance when considering biosecurity. Endophytic or quiescent *Colletotrichum* species may remain undetected and be overlooked during quarantine inspection when citrus fruit or plant material is imported. In addition, *C. citricola* and *C. gloeosporioides* have been observed as saprobes on dead fallen leaves, which may serve as potential sources of inoculum for citrus and other hosts and pose biosecurity risks both locally and during transportation to other regions [42,43,49].

Rapid and accurate identification of *Colletotrichum* species is particularly important in quarantine inspection [10]. Real-time quantitative PCR, which enables the rapid, sensitive, and accurate quantification of target fungal DNA in a wide range of environmental samples, has been developed for several *Colletotrichum* species. De Silva (2019) developed a qPCR assay to differentiate *C. scovillei* from 14 closely related *Colletotrichum* species, especially *C. acutatum* and *C. nymphaeae* that cause anthracnose in chilli [111]. Debode et al. (2009) quantitatively detected *C. acutatum* in naturally infected and symptomless strawberry leaves using real-time PCR [112]. A real-time PCR assay has also been developed for the rapid and accurate identification of *C. coccodes* in symptomatic tomato leaves and external asymptomatic potato tubers [113]. Azevedo-Nogueira et al. (2021) designed a species-specific qPCR assay to enable the rapid detection and quantification of *C. acutatum sensu stricto* in pulverised asymptomatic olive fruit at 16 h post inoculation [114]. Additionally, knowledge of the resistance levels of multiple host varieties to the same pathogen species is essential for breeding resistant varieties for integrated disease management and minimising both economic losses and environmental impacts [24]. Real-time quantitative PCR could also be used to screen resistant host varieties [115,116]. Therefore, real-time quantitative PCR assays could be developed for highly aggressive citrus-associated *Colletotrichum* species and applied in screening procedures for imported produce and in breeding programmes to strengthen plant biosecurity. The problem with developing a PCR diagnostic technique is selecting species-specific primers due to the ever-increasing number of new species, especially closely related pathogenic species.

Globally, records of new *Colletotrichum* species associated with citrus continue to increase, placing greater pressure on plant biosecurity risk management. Accurate identification of species is the initial step, as many species have been isolated solely from one host species in one geographic location. Further studies are urgently needed to provide a more comprehensive understanding of the host ranges and suitable environmental conditions of *Colletotrichum* species. *Colletotrichum* species have shown various levels of aggressiveness and pathotype differences when infecting the same host plant tissue under the same environmental conditions [42,45,48,52,53,80,81,86,102]. The pathogenicity of each *Colletotrichum* species indicates its potential to reduce productivity in citrus orchards. Detached organ assays are useful for rapid screening, require less space than *in planta* assays conducted in greenhouses or fields, and can produce disease symptoms similar to those observed following *in planta* inoculations [48,117]. Therefore, comprehensive pathogenicity bioassays that include common hosts of *Colletotrichum* species and a range of temperature and humidity conditions can be used to determine host range, estimate the potential distribution of newly reported species, and assess their potential to pose biosecurity risks to citrus trees, native plants, and crops.

## Figures and Tables

**Table 2:** Controlled environment bioassays testing the pathogenicity of *Colletotrichum* species associated with *Citrus*.

*Colletotrichum* Species	Tested Host Species	Tested Organ	Inoculation Method (W, NW or NA) ^a^	Inoculum	Analysis Based On	Results Shown As	Reference
*Colletotrichum acutatum*, *C. gloeosporioides* and *C. karstii*	Sweet orange (*Citrus* × *sinensis*) ‘Tarocco Scirè’, lemon (*C. limon*) ‘Femminello 2Kr’ and bergamot (*C. bergamia*) ‘Fantastico’	In planta twig (around 0.5 cm diameter)	W	Mycelium plug (3 mm side)	Lesion length (mm) (14 days post inoculation)	Mean value of each species	[51]
	Lemon ‘Femminello 2Kr’	Green fruitlet	W	Mycelium plug (3 mm side)	Symptom description (3 and 6 days post inoculation)	Value of each isolate	
	Sweet orange ‘Tarocco Meli’ and lemon ‘Femminello 2Kr’	Mature fruit	W and NW	Mycelium plug (3 mm side)	Lesion area (mm^2^) (12 days post inoculation)	Mean value of each species	
	Sweet orange ‘Moro’ and ‘Navelina’	Expanded young and mature leaves	W and NW	Mycelium plug (3 mm side)	Lesion area (mm^2^) (5 days post inoculation)	Value of each isolate	
	Apple (*Malus domestica*) ‘Fuji’ and ‘Cripps Pink’	Mature fruit	W	Mycelium plug (3 mm side)	Lesion area (mm^2^) (7 days post inoculation)	Mean value of each species	
*C. australianum*, *C. fructicola, C. karstii*, *C. novae-zelandiae*, *C. siamense*, *C. theobromicola*, and *C. gloeosporioides*	Lemon (*Citrus* × *limon* ‘Eureka’), Meyer lemon (*Citrus* × *meyeri*) and orange (*Citrus* × *sinensis* ‘Washington Navel’)	Leaf (3 weeks after bud appearance, 4 cm in length lemon and Meyer lemon leaves, 5 cm in length orange leaves)	NW	Spore suspension (6 μL) (10^6^ conidia per ml)	Infection incidence (%) and lesion diameter (mm) (lemon and Meyer lemon leaves were measured 3 and 5 days post inoculation; orange leaves were measured only at 3 days post inoculation)	Value of each isolate	[102]
*C. gigasporum*, *C. kokhaense*, *C. gloeosporioides*, *C. plurivorum*, and *C. siamense*	Lime (*Citrus aurantiifolia* cultivar ‘Pan Pichit’), and tangerine (*C. reticulata* cultivar Sai Nam Phueng)	Mature fruit	W and NW	Mycelial disc (5 mm in diameter)	Disease rating and disease index (2 weeks post inoculation for the orange fruits and 3 weeks post inoculation for the lime fruits)	Value of each isolate	[80]
	Lime cultivars ‘Pan Pichit’ and ‘Nam Phueng’, and tangerine cultivar Sai Nam Phueng	Seedling (6 weeks old)	NW	Mycelial disc (5 mm in diameter)	Disease rating and disease index (4 weeks post inoculation)	Value of each isolate	
	Lime cultivar ‘Pan Pichit’, and tangerine cultivar Sai Nam Phueng	In planta shoot (1 or 2 weeks old)	W	Mycelial disc (5 mm in diameter)	Disease rating (2 months after inoculation for lime and 2.5 months after inoculation for orange)	Value of each isolate	
*C. gloeosporioides*, *C. karstii*, and *C. novae-zelandiae*	Lemon (*Citrus* × *limon* ‘Eureka’)	In planta twig (20 days since bud appearance)	W	Mycelial plug (4 mm^2^)	Symptom description (7 weeks after inoculation)	Value of each isolate	[86]
	Lemon ‘Eureka’ and orange (*Citrus* × *sinensis* ‘Washington Navel’)	Twig (20 days since bud appearance)	NW	Spore suspension (6 μL) (10^6^ conidia per ml)	Infection incidence (%) (lemon twigs recorded at days 4 and 7; orange twigs recorded at day 4)	Value of each isolate	
			W	Spore suspension (6 μL) (10^6^ conidia per ml)	Lesion size (mm) (lemon twigs recorded at days 4 and 7; orange twigs recorded at day 4)	Value of each isolate	
*C. siamense*, *C. gloeosporioides*, *C. fructicola*, *C. endophyticum*, and *C. plurivorum*	Acid lime (*C*. *aurantifolia*)	Mid-ripened fruit	W	Fungal discs (5 mm)	Lesion diameter (mm) (5 to 6 days post inoculation)	Value of each isolate	[45]
	Mandarin (*Citrus reticulata*)	Ripe fruit	W	Fungal discs (5 mm)	Pathogenic or not pathogenic (5 to 6 days post inoculation)	Value of each isolate	
	Acid lime and pomelo (*C*. *maxima*)	Leaf	W	Fungal discs (5 mm)	Pathogenic or not pathogenic (5 to 6 days post inoculation)	Value of each isolate	
	Acid lime	Young branch (around 20 cm in length and 0.5 cm in diameter)	W	Fungal discs (5 mm)	Pathogenic or not pathogenic (14 to 16 days post inoculation)	Value of each isolate	
*C. gloeosporioides* and *C. karstii*	Lemon (*Citrus limon*) cv. Interdonato	Twig, leaf, and fruit	NW	Mycelial plug (5 mm)	Lesion length (mm) (10 days post inoculation)	Value of each isolate	[81]
*C. gloeosporioides*	Apple cv. Gala, pear cv. Santa Maria, banana cv. Cavendish, pepper cv. Capia and avocado cv. Fuerte	Fruit	W	Spore suspension (20 μL) (10^6^ spores per ml)	Lesion length (mm) (10 days post inoculation)	Value of the isolate	
*C. australianum*, *C. fructicola*, *C. gloeosporioides*, *C. karstii*, *C. theobromicola*, *Colletotrichum* sp.	Orange (*Citrus* × *sinensis*) ‘Washington Navel’	Mature fruit	W and NW	Spore suspension (6 μL) (10^6^ conidia per ml)	Infection incidence (%) (10 days post inoculation)	Value of each isolate	[49]
	Orange ‘Washington Navel’ and Meyer lemon (*Citrus* × *meyeri*)	Fully expanded leaf	NW	Spore suspension (6 μL) (10^6^ conidia per ml)	Infection incidence (%) (10 days post inoculation)	Value of each isolate	
	Orange ‘Washington Navel’	Flower petal	NW	Spore suspension (6 μL) (10^3^ conidia per ml)	Infection incidence (%) (3 days post inoculation)	Value of each isolate	
*C. gloeosporioides*, *C. karstii*, and *C. acutatum*	Sweet orange ‘Valencia Late’, mandarin ‘Encore’, lemon ‘Lisbon’ and Key lime	Potted plants (leaf, flower, branch)	NA	Spore suspension (sprayed to runoff) (10^6^ spores per ml)	Infection incidence (%) (10 days post inoculation in leaves, 5 days in flowers and 45 days in branches)	Value of each isolate	[48]
		Flower (closed blossoms)	NW	Spore suspension (20 μL) (10^6^ spores per ml)	Infection incidence (%) (5 days post inoculation)	Value of each isolate	
		Leaf (5 to 7 days old)	W	Spore suspension (20 μL) (10^6^ spores per ml)	Infection incidence (%) (10 days post inoculation)	Value of each isolate	
		Mature fruits	W	Spore suspension (20 μL) (10^6^ spores per ml)	Symptom description (10 days post inoculation)	Only isolates of *C. gloeosporioides*	
*C. gloeosporioides* and *C. karstii*	Orange (*Citrus* *sinensis* (L.) Osbeck) ‘Valencia’ and lemon (*C. limon* L.) ‘Eureka’	Fruit	W	Spore suspension (10 µL) (10^5^ conidia per ml)	Lesion diameter (mm) (7 days after inoculation)	Value of each isolate	[52]
*C. gloeosporioides* and *C. karstii*	*Citrus sinensis* ‘Tarocco Scirè’ and ‘Tarocco Nucellare’	Fruit	W	Spore suspension (10 μL) (10^5^ conidia per ml)	Disease incidence (%) and symptom severity (cm) (15 days after inoculation)	Mean value of each species	[59]
*C. acutatum*, *C. catinaense*, *C. gloeosporioides*, *C. helleniense*, *C. hystricis, C. karstii*, *C. limonicola*, *C. novae-zelandiae*	Sweet orange (*Citrus sinensis*) clones (‘Tarocco Scirè’ and ‘Tarocco Nucellare’)	Fruit	W	Spore suspension (10 μL) (10^5^ conidia per ml)	Infection incidence (%) (10 days after inoculation)	Value of each isolate	[42]
*C. karstii*	Apple (*Malus domestica* (Suckow) Borkh.) cultivar ‘Golden Delicious’	Fruit	W	Spore suspension (20 μL) (10^5^ conidia per ml)	Disease incidence (%) and lesion diameter (cm) (7 days after inoculation)	Value of each isolate	[100]
	Kumquat (*Citrus margarita*) plants grafted to volkamerian lemon (*C. volkameriana* Ten. & Pasq.) rootstock	Twig	W	Mycelium plug (4 mm diameter)	Disease incidence (%) (20 days after inoculation)	Value of each isolate	
*C. gloeosporioides*-like and *C. fructicola*-like	Orange varieties ‘Thomson’, ‘Meski’ and ‘Malti’, lemon variety ‘Lunari’ and sweet lime variety ‘Bergamot’	Fruit	W	Mycelial plug (0.5 cm diameter), and filter paper (0.5 × 0.5 cm) soaked in spore suspension (10^5^ conidia per ml)	Lesion area (cm^2^) (2 days post inoculation)	Value of each isolate	[53]
		Leaf	NA	Filter paper soaked in spore suspension (10^5^ conidia per ml)	Lesion area (cm^2^) (7 to 10 days post inoculation)	Value of each isolate	

Note: Only controlled environment bioassays testing pathogenicity of the same *Colletotrichum* isolate on multiple host species or host tissues, and controlled environment bioassays testing pathogenicity of multiple *Colletotrichum* isolates on the same host tissue are included in Table 2. ^a^ Abbreviations: W, wound inoculated; NW, non-wound inoculated; NA, wound or non-wound inoculation was not mentioned.

## Data Availability

The data that support the findings of this study are available from the corresponding author upon reasonable request.
